# Modulation of Insulin Sensitivity of Hepatocytes by the Pharmacological Downregulation of Phospholipase D

**DOI:** 10.1155/2015/794838

**Published:** 2015-05-24

**Authors:** Nataliya A. Babenko, Vitalina S. Kharchenko

**Affiliations:** Department of Physiology of Ontogenesis, Biology Research Institute, Karazin Kharkov National University, Svobody Square 4, Kharkov 61022, Ukraine

## Abstract

*Background*. The role of phospholipase D (PLD) as a positive modulator of glucose uptake activation by insulin in muscle and adipose cells has been demonstrated. The role of PLD in the regulation of glucose metabolism by insulin in the primary hepatocytes has been determined in this study. *Methods*. For this purpose, we studied effects of inhibitors of PLD on glucose uptake and glycogen synthesis stimulation by insulin. To determine the PLD activity, the method based on determination of products of transphosphatidylation reaction, phosphatidylethanol or phosphatidylbutanol, was used. *Results*. Inhibition of PLD by a general antagonist (1-butanol) or specific inhibitor, halopemide, or N-hexanoylsphingosine, or by cellular ceramides accumulated in doxorubicin-treated hepatocytes decreased insulin-stimulated glucose metabolism. Doxorubicin-induced hepatocytes resistance to insulin action could be abolished by inhibition of ceramide production. Halopemide could nullify this effect. Addition of propranolol, as well as inhibitors of phosphatidylinositol 3-kinase (PI3-kinase) (wortmannin, LY294002) or suppressors of Akt phosphorylation/activity, luteolin-7-O-glucoside or apigenin-7-O-glucoside, to the culture media could block cell response to insulin action. *Conclusion*. PLD plays an important role in the insulin signaling in the hepatocytes. PLD is activated downstream of PI3-kinase and Akt and is highly sensitive to ceramide content in the liver cells.

## 1. Introduction

Phospholipase D (PLD) has been implicated in the generation of phosphatidic acid (PA). PA serves as a critical lipid second messenger that regulates several proteins implicated in the regulation of cellular metabolism, cell cycle progression, and cell growth [1]. The PLD pathway is activated in response to stress, growth factors, and insulin. PLD exhibits various effects on mammalian target of rapamycin (mTOR) signaling, which plays a critical role in insulin signal transduction. PA derived from the phospholipids hydrolysis activates mTORC1 and mTORC2, each of them having distinct effects on insulin action in the target tissues. However, PA derived from PLD1, unlike* de novo* synthesized PA in mice hepatocytes, influences Akt phosphorylation through a mechanism other than the mTOR/rictor assembly [2]. Insulin stimulates the protein kinase C- (PKC-) and phospholipase C*γ*-dependent activity of the PLD in rat hepatocytes and HEK 293 cells [3, 4]. In rat adipocytes, the insulin-induced activation of phosphatidylinositol-3-kinase (PI3-kinase) leads to production of the polyphosphoinositides in the plasma membrane with a subsequent translocation of Rho and activation of the PLD [5].

PLD/PA regulates different steps of vesicle trafficking, including activation of signaling networks [6], budding of vesicles from the trans-Golgi [7], and vesicle fusion [8]. The PLD-dependent generation of PA, followed by an activation of phosphatidylinositol 4-phosphate 5-kinase [9], increases the levels of a phosphatidylinositol 4,5-bisphosphate critically required for exocytosis [10]. PA can be converted by phosphatidate phosphohydrolase to diacylglycerol, which itself is a highly fusogenic lipid [11]. Along with the atypical PKC forms *ζ* and *λ* and protein kinase B, the PLD is involved in insulin regulation of the key stage of glucose transport, particularly, the translocation of glucose transporters (Glut) from the endoplasmic reticulum into the plasma membrane [12]. Overexpression of human PLD1 facilitates the insulin-stimulated Glut4 trafficking and glucose uptake. Interference with PLD1 function, via expression of a catalytically inactive allele or via selective loss of PLD1 through RNAi targeting, leads to insulin resistance with respect to Glut4 trafficking and glucose uptake [13]. The role of the PLD1-generated PA in the final steps of Glut4-containing vesicle fusion into the plasma membrane in response to insulin has been reported [13]. Recently it has been demonstrated that PLD may mediate insulin effects on fusion pore stability in the 3T3-L1 adipocyte [14]. Chronic depletion of PLD1 caused vesicles to be “docked” at the plasma membrane in response to insulin, but not to be fused [13], while an acute inhibition of the PLD pathway with a general antagonist (1-butanol) or a new inhibitor (5-fluoro-2-indolyl des-chlorohalopemide (FIPI)) increased the frequency of kiss-and-run events in basal and insulin-stimulated cells [14]. These results implicate the PLD in the regulation of fusion pore dynamics in the 3T3-L1 adipocytes. Insulin lowers the barrier to full vesicle fusion via the PLD and thus reduces the kiss-and-run frequency. Moreover, other important cellular targets of PLD/PA in the insulin signaling machinery have been identified in liver and other tissues [15, 16]. The atypical protein kinases C, PKC-*ζ* and PKC*λ*, are prime targets for* de novo* synthesized PA and play a critically important role in the development of impaired glucose metabolism, systemic insulin resistance, and excessive hepatic production of glucose, lipids, and proinflammatory factors. The unique interaction between PA and PKC-*ζ*, which is not found for other PKC isotypes, has been found in the COS cells, which are overexpressing atypical PKC [17]. The PKC*λ* is of special importance among the various metabolic actions of insulin [18] as it contributes to induction of the expression of Srebp1c and genes of triglyceride synthesis in the liver. Moreover, insulin resistance is often associated with increases both in triglyceride content and in the expression of Srebp1c in the liver cells.

Although insulin receptors as well as Gluts [19] and insulin activated PLD [4, 20] are present in hepatic cells, the impact of PLD in the regulation of glucose metabolism by insulin in the hepatocytes is still obscure. It has been found that the age- and ceramide-dependent insulin resistance of rat hepatocytes coincides with a reduction of insulin-induced PLD activation, glucose uptake by the cells, and glycogen synthesis [20]. The PLD as well as glucose metabolism stimulation by insulin in the insulin-resistant hepatocytes can be improved with the inhibitors of ceramide synthesis* de novo* and sphingomyelin (SM) degradation [20, 21].

We showed in the present study that insulin activated the PLD in the different tissues. Among the insulin targets there were liver, muscles, neocortex, and kidney. Induction of PLD by insulin was coincident with activation of glucose uptake and glycogen synthesis in the liver cells. A general antagonist of the PLD/PA pathway, 1-butanol, or a specific inhibitor of PLD, halopemide, reduced activation of PLD by insulin, as well as insulin-induced glucose metabolism in the hepatocytes. Similar results were obtained under the action of the PA phosphohydrolase inhibitor, propranolol, or PI3-kinase inhibitors, wortmannin and LY294002, on the liver cells. The C6-ceramide addition to the culture media or induction of ceramide production with doxorubicin reduced not only the PLD activation by insulin but hormone-induced glucose uptake and glycogen synthesis in the hepatocytes, too. Drug-induced inhibition of insulin action in the PLD activity and glucose metabolism could be avoided with the inhibitors of specific pathways of ceramide production in the cells. Halopemide could nullify this effect. Addition to the culture media known suppressors of Akt phosphorylation/activity, luteolin-7-O-glucoside (LU7Glu) or apigenin-7-O-glucoside (AP7Glu), resulted in inhibition of PLD activity and glucose metabolism in the insulin-stimulated cells. Results obtained have demonstrated that in the insulin-stimulated hepatocytes the PLD/PA pathway was activated downstream of PI3-kinase and possibly Akt. The PLD is highly sensitive to the ceramide level in the cells and is a crucial factor for the stimulation of glucose metabolism by insulin in hepatocytes.

## 2. Methods

### 2.1. Animals

The 90-day-old male Wistar rats weighing 220–250 g were used in the experiments. They were kept at 24°C on a cycle of 12 h light/12 h darkness and had a free access to a standard chow diet and drinking water ad libitum. All experiments on animals were carried out according to the International Principles of the European Convention for the Protection of Vertebrate Animals Used for Experimental and Other Scientific Purposes (Strasbourg, 1985) and National General Ethical Principles for Experiments on Animals (Ukraine, 2001). Experimental procedures were approved by the Institutional Animal Care and Use Committees at the Kharkov Karazin National University. Rats were fasted overnight and were anesthetized the next day with an injection of ketamine (75 mg/kg) and sacrificed by decapitation. Their tissues (liver, skeletal muscle soleus, brain cortex, kidney cortex) were used for determination of PLD activity. Hepatocytes were isolated from livers as described below.

### 2.2. Experiments with Tissues Slices

Slices of liver, muscle, brain, and kidney cortex were incubated in the Krebs-Ringer bicarbonate buffer, pH 7.4, in the presence of [^14^C]palmitic acid (0,25 *µ*Ci/mL) (56 mCi/mmol, Amersham, GE Health Care, UK) for 1.5 h at 37°C in the atmosphere of 5% CO_2_ and 95% O_2_. Tissues slices prelabeled with [^14^C]palmitic acid were used to determine the insulin effects on the PLD activity as described below.

### 2.3. Experiments with Isolated Hepatocytes

Hepatocytes were isolated as described in [22]. The cell viability was estimated using Trypan Blue. The cell survival was 90–96%. Freshly isolated hepatocytes were resuspended in Eagle medium (Institute of Poliomyelitis and Viral Encephalitis, Russia) containing 10% fetal serum (BioloT, Russia), 20 mM HEPES, penicillin (61 mg/liter), streptomycin (100 mg/liter), 4·10^7^ cells/mL and then incubated for 2 h at 37°C in the presence of [^14^C]palmitic acid (0.25 *µ*Ci/mL) (Amersham, GE Health Care, UK). To study the effect of general antagonist of PLD on insulin signaling, the liver cells were treated with 10 nM insulin (monocomponent porcine insulin; Indar, Ukraine) or saline (0,9% NaCl) and incubated for 5 min at 37°C with a subsequent addition of 0,1 % 2-butanol (as control) or 0,1% 1-butanol to the cell suspension and hepatocytes incubation for 5 and 30 min. The reaction was stopped with ice-cold mixture of chloroform : methanol (1 : 2) and the PLD activity was determined as described below. For determination of butanol effects on the insulin-stimulated glucose metabolism, the hepatocytes, not labeled with [^14^C]palmitic acid, were used. To study the effect of different inhibitors of the PLD/PA pathway on insulin signaling, the hepatocytes prelabeled with [^14^C]palmitic acid (for PLD activity determination) and the cells not labeled with [^14^C]palmitic acid (for determination of glucose metabolism) were treated with a specific PLD inhibitor, halopemide (200, 300 nM) (Sigma, USA) or N-hexanoylsphingosine (C6-ceramide) (Sigma, USA) for 90 min or propranolol (100 *µ*M) (Sigma, USA) for 15 min. To clarify the impact of PI3-kinase on insulin-stimulated PLD and glucose metabolism, the specific inhibitors of PI3-kinase were used. The hepatocytes, prelabeled with [^14^C]palmitic acid (for PLD activity determination), and cells, not labeled with [^14^C]palmitic acid (for determination of glucose metabolism), were treated with wortmannin (100 nM) (Sigma, USA) or LY294002 (100 nM) (Sigma, USA) or DMSO (control). For modulation of endogenous ceramides contents in the hepatocytes and its sensitivity to insulin action the cells were incubated in the presence of [^14^C]sodium acetate (24,5 *µ*Ci/mL) and doxorubicin (30 nM) (Pharmachemie B.V., Netherlands) or the mixture of doxorubicin (30 nM) and myriocin (5 *µ*M), or doxorubicin (30 nM) and GW4869 (20 *µ*M) (Sigma-Aldrich, Germany), or doxorubicin (30 nM) and imipramine (50 *µ*M) (Sigma, USA) or doxorubicin (30 nM) and the mixture of total inhibitors for 90 min. The control plates contained appropriate amounts of solvents for inhibitors. The cells, incubated with different inhibitors and modulators, subsequently treated with insulin (10 nM) or 0,9% NaCl (control) for 5 and 30 min were used for determination of PLD activity and glucose metabolism as described below. To study effect of LU7Glu and AP7Glu on PLD activity the isolated hepatocytes were labeled with [^14^C]sodium acetate (24,5 *µ*Ci/mL) for 90 min and then treated with insulin (10 nM) or 0,9% NaCl (control) for 5 min and subsequently with LU7Glu (20 *µ*M) or AP7Glu (20 *µ*M) (State Scientific Center of Drugs, Kharkov, Ukraine) or DMSO (control) for 15 min. After that 0,1% ethanol added to the culture media and cells were incubated for 20 min. Additionally, the hepatocytes, prelabeled with [^14^C]sodium acetate, were treated for 15 min with LU7Glu (20 *µ*M) or AP7Glu (20 *µ*M) before insulin (10 nM) or 0,9% NaCl (control) and 0,1% ethanol addition to the incubation media. Cells treated by flavones and insulin were used for determination of PLD activity as described below. For determination of flavones effect on glucose metabolism in the insulin-stimulated cell the hepatocytes, not labeled with [^14^C]sodium acetate, were used, and glucose uptake and glycogen synthesis were studied as described below.

### 2.4. Determination of Phospholipase D Activity

To determine the PLD activity in rat tissues and isolated hepatocytes, a sensitive assay was used. The method was based on formation of phosphatidylethanol (PET) or phosphatidylbutanol (PBUT), which were produced only by PLD as a result of transphosphatidylation in the presence of ethanol or butanol [23–26]. Once synthesized, the PET and PBUT metabolized very slowly and, therefore, could be used as an indicator of PLD activation in stimulated cells. To determine the PLD activity, the prelabeled with [^14^C]palmitic acid tissues slices or isolated hepatocytes were washed in Krebs-Henseleit buffer with c 0.1% BSA and diluted in the same buffer to the cell concentration of 2·10^7^ cell/mL. Before addition of insulin into the incubation medium, the cells were preincubated in the presence of 300 mM ethanol or 300 mM butanol for 10 min, and then 10 nM insulin or 0.9% NaCl (as a control for insulin) was added to the medium. The reaction was stopped within 5 or 30 min. Lipids were extracted and analyzed as described in [26]. The gel spots containing [^14^C]PET, [^14^C]PBUT, and [^14^C]phosphatidylcholine (PC) were scraped and transferred to scintillation vials. Radioactivity was measured by a scintillation counter.

### 2.5. Determination of Glucose Metabolism

Non-^14^C-labeled hepatocytes were used to study insulin-induced uptake of 2-D-[^3^H]glucose (0.5 *μ*Ci/mL) and incorporation of D-[U ^14^C]glucose (0.1 *μ*Ci/mL) into glycogen by the method of Brutman-Barazani et al. [27]. Cells were washed free from the additions in the HBS buffer (HEPES-buffered saline) containing 20 mM HEPES. Then hepatocytes were incubated in the same buffer for 30 min in the presence of 10 nM insulin (or 0.9% NaCl as a control) and then were washed by the HBS buffer and diluted in the buffer, supplemented with 0.5 *μ*Ci/mL of 2-D-[^3^H]glucose, and incubated for 10 min at 37°C. To determine glycogen synthesis, the washed cells were incubated in HBS buffer in the presence of 5 mM glucose, 10 nM insulin or 0.9% NaCl (control) and 0.1 *μ*Ci/mL of D-[U ^14^C]glucose for 2 h at 37°C. The reaction was stopped with ice-cold 0.9% NaCl and the hepatocytes were washed with the same solution three times. The cells were lysed with 50 mM NaOH. Radioactivity of the ^3^H-glucose and ^14^C-glycogen was measured using a BETA scintillation counter.

### 2.6. Extraction and Separation of Lipids

The lipids were extracted according to the Bligh and Dyer protocol [28]. The chloroform phase was collected and dried under N_2_ at 37°C. The lipids were redissolved in chloroform/methanol (1 : 2, v/v) and applied on TLC plates. To separate [^14^C]ceramide, the plates with loaded lipid extract were developed with chloroform/ethylacetate/propanol/methanol/0.25% KCl (25 : 25 : 25 : 10 : 9, v/v). The appropriate standards were applied on each plate for quantification. The Rf value for ceramide was 0.90. The regions migrating with the standard ceramide were scraped from the plate and transferred to scintillation vials. Radioactivity was measured by a scintillation counter. Content of protein in the samples was determined according to Lowry et al. [29].

### 2.7. Statistical Analysis

Data were analyzed by one-way analysis of variance (ANOVA) followed by post hoc Fisher's protected least significant difference (Fisher PLSD) test. The results obtained represent the means ± standard error of the mean (SEM) and are deemed statistically significant when *p* < 0.05. The statistical analysis was carried out with StatSoft Statistica v6.0.

## 3. Results

### 3.1. Effect of Insulin on Phospholipase D Activity in Different Rat Tissues

**Figure 1:**
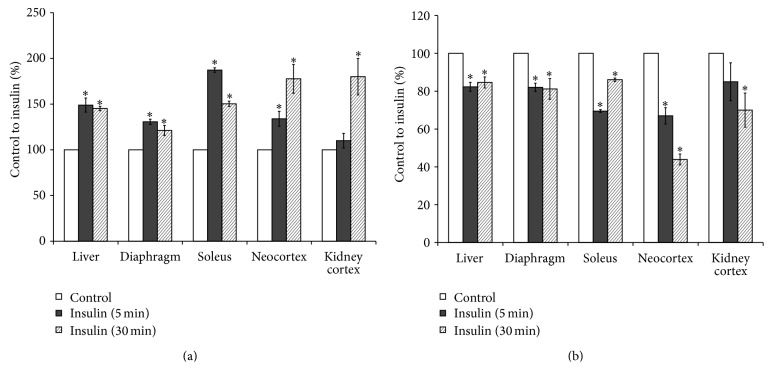
Effect of insulin on phospholipase D activity in rat tissues slices. (a) [^14^C]phosphatidylethanol, (b) [^14^C]phosphatidylcholine. Formation of [^14^C]phosphatidylethanol and degradation of substratum of phospholipase D, [^14^C]phosphatidylcholine in the presence of 300 mM ethanol and 10 nM insulin was determined as described in the Methods. ^*∗*^ *p* < 0.05, insulin versus control.

**Figure 2:**
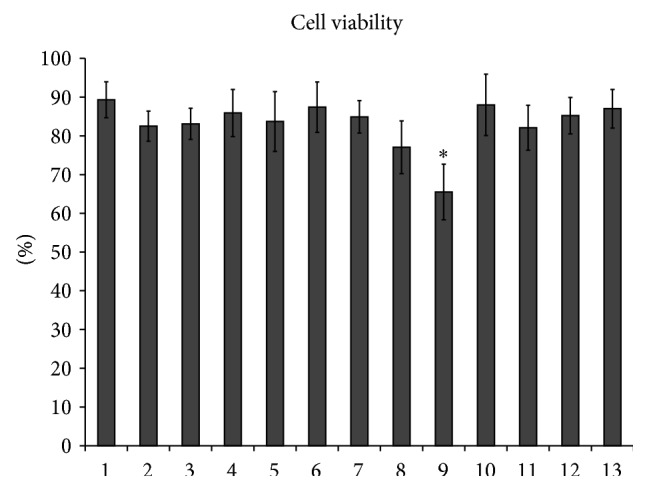
Effects of alcohol and drugs on hepatocyte viability. 1: control cells, 2: ethanol, 3: butanol, 4: wortmannin, 5: LY294002, 6: propranolol, 7: halopemide, 8: C6-ceramide, 9: doxorubicin, 10: myriocin, 11: imipramine, 12: GW4869, 13: total sphingolipid turnover inhibitors. ^*∗*^ *p* < 0.05, drug versus control.

For determination of insulin-stimulated endogenous PLD activation in rat tissues a sensitive assay was used. The method was based on formation of phosphatidylethanol (PET) or phosphatidylbutanol (PBUT), which can be produced only by PLD as a result of transphosphatidylation in the presence of ethanol or butanol [23–26]. Our results have shown that addition of insulin to the culture medium of liver, muscle, brain, and kidney cortex slices significantly increased activity of PLD. Insulin stimulated the [^14^C]PET production (Figure 1(a)) and decreased the content of PLD substrate, [^14^C]PC (Figure 1(b)), in all studied tissues prelabeled with [^14^C]palmitic acid. These results and data previously obtained on isolated hepatocytes [4, 20] have demonstrated that insulin rapidly activated PC-specific PLD in the classical insulin responsive tissues (liver, muscle) as well as in the brain and kidney. Taking into account the fact that the alcohol substrate, required to detect the PLD activity, can be toxic for the cells, we studied the liver cell viability using Trypan Blue. The short-term ethanol or butanol treatment of isolated hepatocytes had no significant effect on the cell survival (Figure 2).

### 3.2. Effect of Phosphoinositide-3-Kinase Inhibitors on Phospholipase D and Glucose Metabolism Stimulation by Insulin

**Figure 3:**
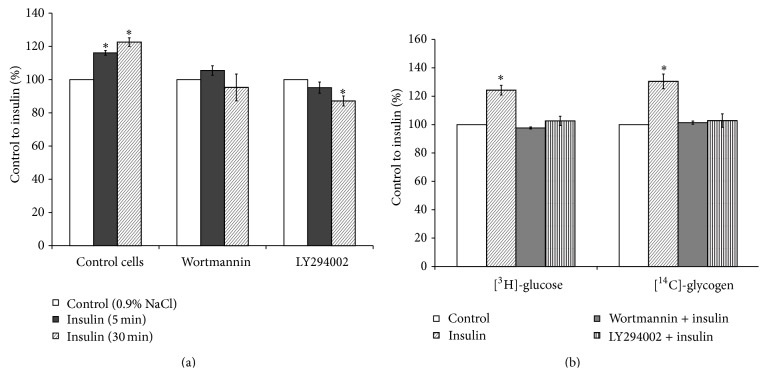
Effects of inhibitors of phosphoinositide-3-kinase on phospholipase D activity and glucose metabolism in insulin-stimulated hepatocytes. (a) Phospholipase D activity, (b) uptake of 2-D-[^3^H]glucose and incorporation of D-[U ^14^C]glucose into glycogen. Effect of insulin on PLD activity and glucose metabolism in the wortmannin- or LY294002-treated hepatocytes was determined as described in the Methods. ^*∗*^ *p* < 0.05, insulin versus control.

Product of PI3-kinase, phosphatidylinositol 3,4,5-trisphosphate (PIP3), is the main second messenger for insulin and mediates most of its metabolic actions in the target tissues. The findings obtained on adipocytes, treated with wortmannin, suggested that the effect of insulin on phospholipase D-mediated hydrolysis of PC is largely dependent upon PI3-kinase activation [30]. Furthermore, insulin also activates cPKCs and nPKCs, particularly in liver and adipose tissues, by activating* de novo* synthesis of PA and these effects are not dependent on the PI3-kinase [15]. In order to clarify if the PI3-kinase mediated insulin influence on PLD activity in the hepatocytes, two different specific inhibitors of PI3-kinase, wortmannin and LY294002, were used in the present study. Addition of wortmannin or LY294002 to the culture media of hepatocytes had no effect on cell viability (Figure 2) and nullified stimulatory effect of insulin on the [^14^C]PET production (Figure 3(a)), as well as on the [^3^H]glucose uptake and [^14^C]glycogen synthesis (Figure 3(b)).

### 3.3. Effect of Acute Perturbation of Phospholipase D with 1-Butanol on Glucose Metabolism Stimulation by Insulin

**Figure 4:**
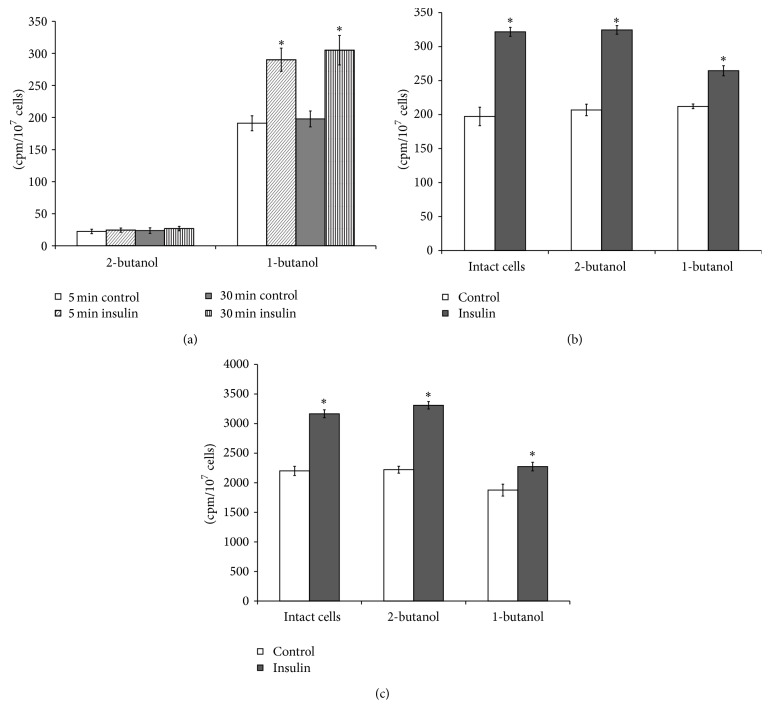
Effects of 1-butanol on phospholipase D activity and glucose metabolism stimulation by insulin in the isolated hepatocytes. (a) Phospholipase D activity, (b) uptake of 2-D-[^3^H]glucose, (c) incorporation of D-[U ^14^C]glucose into glycogen. Effect of insulin on phospholipase D activity and glucose metabolism in the 1-butanol- or 2-butanol-pretreated hepatocytes was determined as described in the Methods. ^*∗*^ *p* < 0.05, insulin versus control; ^*∗∗*^ *p* < 0.05, insulin + 1-butanol versus insulin + 2-butanol; ^*∗∗∗*^ *p* < 0.05, 1-butanol versus 2-butanol.

As depletion of PLD could modulate insulin signaling, we tested if acute perturbation of PLD with a general antagonist (1-butanol) affected the insulin-induced activation of glucose uptake and glycogen synthesis in hepatocytes. Addition of 1-butanol to the culture media resulted in increase of [^14^C]PBUT formation in the insulin-treated cells. At the same time, 2-butanol (control) addition to the prelabeled with [^14^C]palmitic acid hepatocytes practically was not followed by the [^14^C]PBUT synthesis in the insulin-treated and hormone untreated cells (Figure 4(a)). However, 2-butanol did not change the stimulatory effect of insulin on [^3^H]glucose uptake by the cells (Figure 4(b)) and [^14^C]glycogen synthesis (Figure 4(c)), while 1-butanol reduced activation of glucose metabolism by insulin in hepatocytes (Figures 4(b) and 4(c)). These data implicate PLD/PA in the regulation of glucose uptake and glycogen synthesis in the hepatocytes.

### 3.4. Effect of Propranolol on Phospholipase D Activity and Glucose Uptake in the Insulin-Stimulated Liver Cells

**Figure 5:**
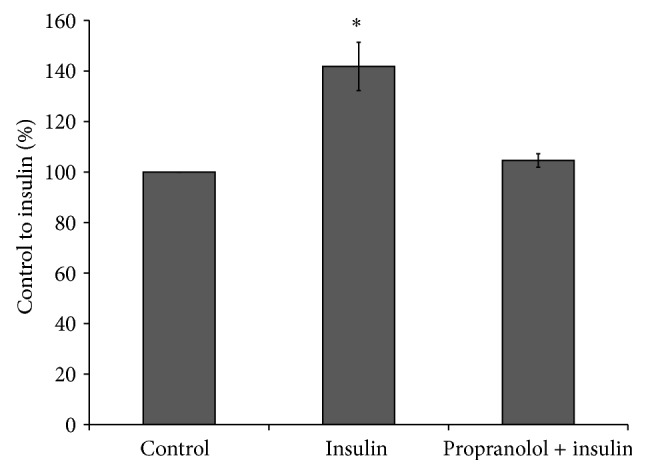
Effect of propranolol on insulin-stimulated glucose uptake by isolated hepatocytes. Insulin-stimulated glucose uptake and propranolol treatments were done as described in the Methods. ^*∗*^ *p* < 0.05, insulin versus control.

Propranolol is a *β*-adrenergic receptor blocking agent. It also blocks the DAG formation by inhibiting the phosphatidate phosphohydrolase. Propranolol is a useful tool to clarify the role of PLD/PA in hormones signaling pathways. As our results showed, addition of propranolol to the culture media prior to insulin did not change the hepatocytes viability (Figure 2) but significantly reduced the [^14^C]PET, as well as [^14^C]DAG formation in the liver cells. The [^14^C]PET contents in insulin- and insulin + propranolol-treated cells were 9537 ± 625 and 5504 ± 327 cpm/10^7^ cells (^*∗*^ *p* < 0.05, insulin + propranolol versus insulin), respectively. The [^14^C]DAG contents in insulin- and insulin + propranolol-treated cells were 3925 ± 642 and 1936 ± 178 cpm/10^7^ cells (^*∗*^ *p* < 0.05, insulin + propranolol versus insulin), respectively. Furthermore, propranolol nullified stimulatory effect of insulin on [^3^H]glucose uptake by hepatocytes (Figure 5).

### 3.5. Effect of Inhibitors of Phospholipase D Activity on Stimulation of Glucose Metabolism by Insulin

**Figure 6:**
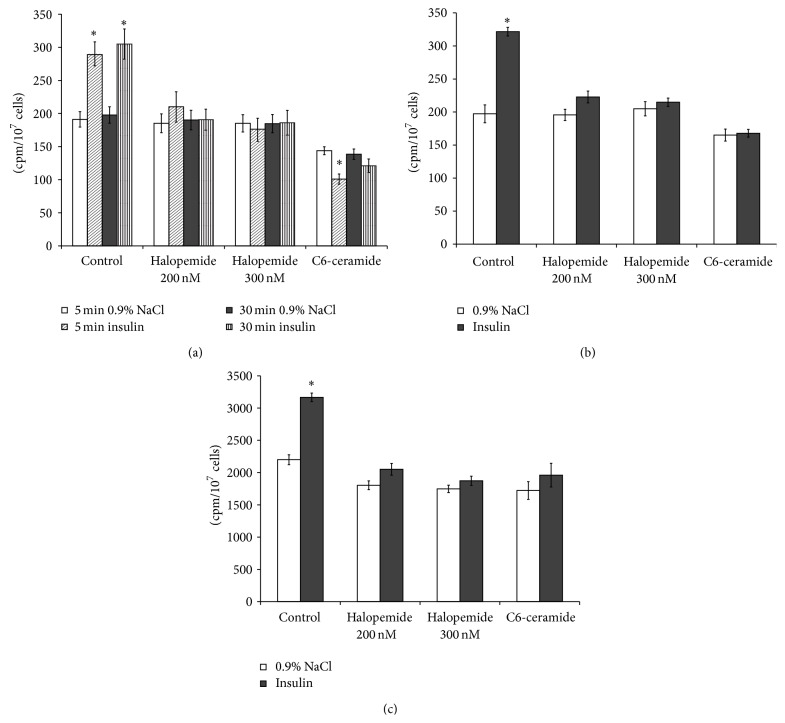
Effect of halopemide and ceramide on phospholipase D activity and glucose metabolism stimulation by insulin in the isolated hepatocytes. (a) Phospholipase D activity, (b) uptake of 2-D-[^3^H]glucose, (c) incorporation of D-[U ^14^C]glucose into glycogen. Effect of insulin on phospholipase D activity and glucose metabolism in the halopemide or C6-ceramide-pretreated hepatocytes was determined as described in the Methods. ^*∗*^ *p* < 0.05, insulin versus control; ^*∗∗*^ *p* < 0.05, insulin + drug versus insulin; ^*∗∗∗*^ *p* < 0.05, C6-ceramide in basal condition versus basal condition, control cells.

PLD1 and PLD2 are expressed in a variety of cell types. Both PLDs could be activated by insulin in the target tissues [3, 13, 31, 32]. To study further the involvement of PLD in insulin signaling in the liver cells, we used the specific inhibitor of PLD, halopemide [33, 34], and a well known inhibitor of agonist-stimulated PLD activity, ceramide [35, 36]. Halopemide and all of the halopemide analogues are described as dual PLD1/2 inhibitors [37]. Pretreatment of hepatocytes with halopemide in concentrations 200 and 300 nM abolished insulin-stimulatory effect on PLD activity (Figure 6(a)) and significantly reduced the induction of the [^3^H]glucose uptake (Figure 6(b)) and [^14^C]glycogen synthesis by insulin (Figure 6(c)). However, treatment of rat hepatocytes with halopemide did not change cells viability, estimated by using Trypan Blue (Figure 2) as well as the basal activity of PLD in the hepatocytes (Figure 6(a)). Halopemide did not affect the [^3^H]glucose uptake (Figure 6(b)) and [^14^C]glycogen synthesis (Figure 6(c)) in the liver cells incubated without insulin. In contrast, addition of the C6-ceramide to the culture media, which is known to selectively decrease the activity and expression of the PLD1 [38], reduced the basal PLD activity (Figure 6(a)), [^3^H]glucose uptake (Figure 6(b)), and [^14^C]glycogen synthesis in hepatocytes (Figure 6(c)). The C6-ceramide abolished stimulatory effect of insulin on PLD activity (Figure 6(a)), [^3^H]glucose uptake (Figure 6(b)), and [^14^C]glycogen synthesis in hepatocytes (Figure 6(c)). The short-term treatment of hepatocytes with C6-ceramide did not change the cells viability (Figure 2).

### 3.6. Effect of Doxorubicin on Stimulation of Phospholipase D and Glycogen Synthesis by Insulin

Doxorubicin is an anticancer drug and a well known stimulator of sphingolipid metabolism in the cells. Doxorubicin induces ceramide accumulation in rat hepatocytes [39] and other types of the cells [40, 41]. Moreover, doxorubicin can decrease expression of genes (IRS1, Glut4, AMPK, and GSK3b) involved in insulin signaling in muscle tissues and thus can cause systemic insulin resistance [42], thus leading to the development of type 2 diabetes-like condition [43].

**Figure 7:**
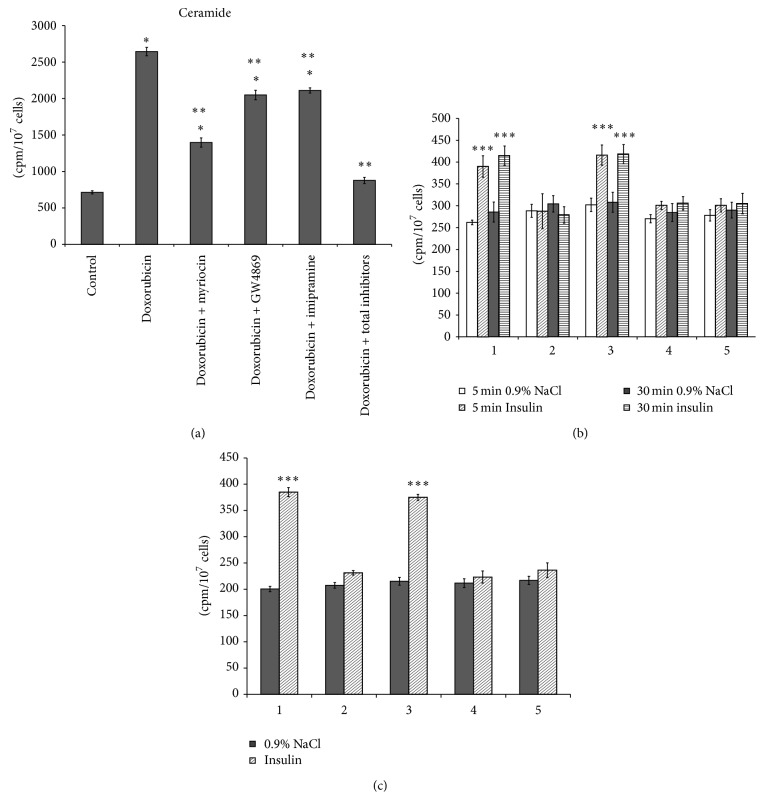
Effect of doxorubicin and inhibitors of sphingolipid turnover and phospholipase D activity on ceramide content and glucose metabolism stimulation by insulin in the isolated hepatocytes. (a) [^14^C]ceramide, (b) uptake of 2-D-[^3^H]glucose, (c) incorporation of D-[U ^14^C]glucose into glycogen. 1: cells treated with DMSO (control cells); 2: doxorubicin; 3: doxorubicin + total sphingolipid turnover inhibitors; 4: doxorubicin + total sphingolipid turnover inhibitors + halopemide (200 nM); 5: doxorubicin + total sphingolipid turnover inhibitors + halopemide (300 nM). Effect of insulin on phospholipase D activity and glucose metabolism in drug-pretreated hepatocytes was determined as described in the Methods. ^*∗*^ *p* < 0.05, doxorubicin versus control; ^*∗∗*^ *p* < 0.05, doxorubicin + drug versus doxorubicin; ^*∗∗∗*^ *p* < 0.05, insulin versus control.

To study the effect of endogenous ceramide on stimulation of PLD and glucose metabolism by insulin, the hepatocytes were pretreated with doxorubicin prior to insulin addition to the culture media. The doxorubicin was determined to increase the liver cell viability (Figure 2) and significantly enhance the ceramide level in the hormone-untreated cells (Figure 7(a)). Doxorubicin nullified the stimulatory effect of insulin on the PLD activity (Figure 7(b)), [^3^H]glucose uptake and [^14^C]glycogen synthesis in hepatocytes (Figure 7(c)). Taking into account the fact that doxorubicin stimulates different pathways of sphingolipid turnover in the next set of experiments, we used different inhibitors of sphingolipid metabolism to abolish the drug-induced ceramide accumulation in the hepatocytes. Inhibition of key enzymes of sphingolipid synthesis (serine palmitoyl transferase) and SM degradation (neutral and acid SMases) under action of specific inhibitors, myriocin [44], GW4869 [45], and imipramine [46], partly prevented ceramide accumulation in the doxorubicin-treated hepatocytes (Figure 7(a)). However, ceramide content can be improved when using all inhibitors of sphingolipid turnover on hepatocytes, as has been done in the present paper. Normalization of ceramide level in the doxorubicin-treated cells by means of the “cocktail” of inhibitors restored hepatocytes responses to insulin action. Addition of all inhibitors of key enzymes of ceramide synthesis (myriocin) and ceramide production from SM (GW4869, imipramine) to the culture media increased activation of PLD (Figure 7(b)), [^3^H]glucose uptake and [^14^C]glycogen synthesis by insulin in the hepatocytes (Figure 7(c)). Addition of myriocin or imipramine or GW4869 or “cocktail” of inhibitors to the culture media nullified doxorubicin effect on hepatocytes viability (Figure 2). The results obtained demonstrated that response of hepatocytes to insulin action could be changed by modulation of exogenous ceramide levels via stimulation or inhibition of sphingolipids turnover. Moreover, addition of the inhibitor of PLD activity, halopemide, to the culture media prior to the “cocktail” of inhibitors of sphingolipids turnover reduced the insulin-stimulated [^3^H]glucose uptake and [^14^C]glycogen synthesis in the hepatocytes (Figure 7(c)).

### 3.7. Effect of Modulators of Insulin Signaling on Stimulation of Phospholipase D and Glucose Metabolism by Insulin

**Figure 8:**
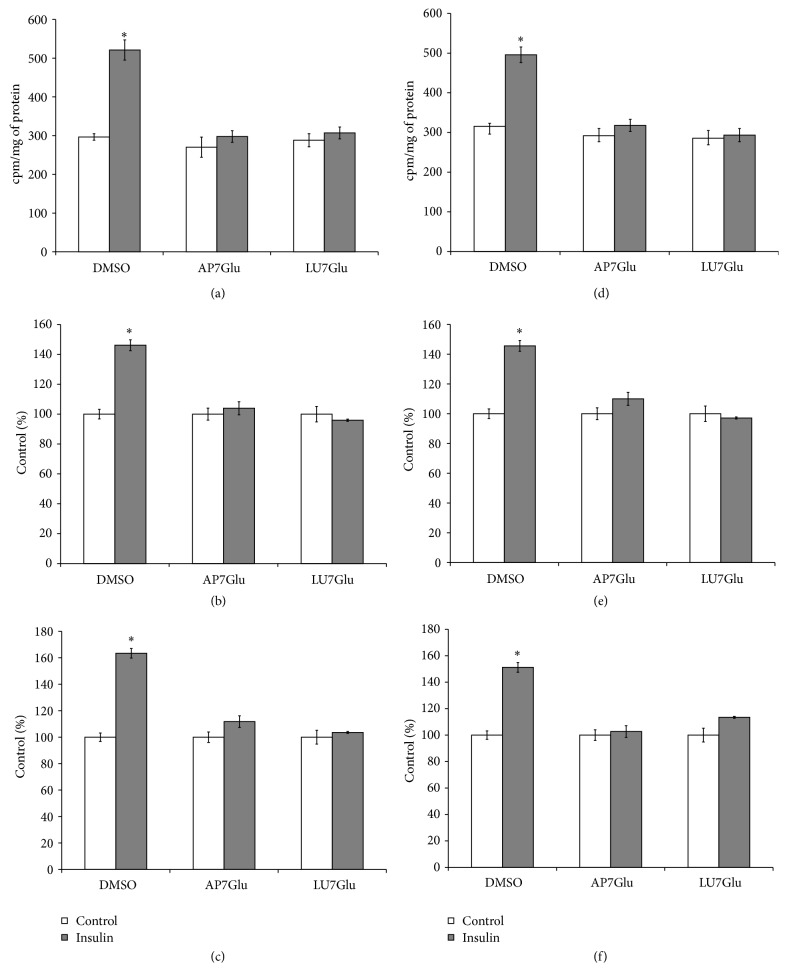
Effect of apigenin-7-O-glucoside and luteolin-7-O-glucoside on phospholipase D activity and glucose metabolism stimulation by insulin in the isolated hepatocytes. Apigenin-7-O-glucoside—AP7Glu, luteolin-7-O-glucoside—LU7Glu. Flavones added to the culture media prior to (a, b, c) and after (d, e, f) insulin as described in the Methods. (a, d) Phospholipase D activity, (b, e) uptake of 2-D-[^3^H]glucose, (c, f) incorporation of D-[U ^14^C]glucose into glycogen. ^*∗*^ *p* < 0.05, insulin versus control.

Luteolin and apigenin and their glycosidic forms are well known modulators of insulin signaling in the target cells. Both flavones inhibit insulin-stimulated Akt phosphorylation/activity, Glut4 translocation into the plasma membrane, and glucose uptake [47, 48]. In the present work, we studied effects of LU7Glu and AP7Glu on PLD activity and glucose uptake and glycogen synthesis in the rat hepatocytes, stimulated by insulin. Taking into account the fact that luteolin inhibits insulin-stimulated phosphorylation of insulin receptor- (IR-) *β* subunit, and apigenin only tended to inhibit the IR-phosphorylation [47], in the present study we performed experiments of two types: (1) flavones were added to the incubation media prior to insulin; (2) flavones were added after insulin addition. Trypan Blue staining indicated that there was no disruption of hepatocytes membranes under LU7Glu or AP7Glu action (data not shown). These results are in line with a previous work on hepatocytes treated with LU7Glu or AP7Glu [49, 50]. Addition of flavones to the incubation media prior to (Figures 8(a), 8(b), and 8(c)) or after (Figures 8(d), 8(e), and 8(f)) insulin was accompanied by significant reduction of insulin-stimulated PLD activity, as well as glucose uptake and glycogen synthesis in rat hepatocytes, while flavones did not alter the PLD activity and glucose metabolism in the nontreated cells. No difference was found between these two types of experiments. These results suggest that both drugs, LU7Glu and AP7Glu, act mainly downstream of IR and are potent suppressors of insulin-stimulated PLD and glucose metabolism in the liver cells.

## 4. Discussion

Liver is a target for insulin action and plays an extremely important role in glucose homeostasis regulation in organism. Using the liver insulin receptor knockout (LIRKO) mouse as a model of pure hepatic insulin resistance it has been demonstrated that hepatic insulin resistance alone can produce both the dyslipidemia and increasing risk of atherosclerosis associated with the metabolic syndrome [51]. The mice used developed hyperinsulinemia, but their livers were unable to respond to hormone action. Inactivation of the insulin receptor gene only in the hepatocytes in LIRKO mice resulted in a complete loss of early insulin signaling events, such as IRS1/2 phosphorylation, glycogen storage, and suppression of glucose production [52]. Furthermore, liver insulin resistance may promote *β* cell hyperplasia observed in type 2 diabetes.

In the hepatocytes, insulin normally acts through cell-surface receptors suppressing glycogenolysis and gluconeogenesis and activating glycogen synthesis and lipogenesis. This signaling pathway, including insulin receptor, IRS proteins, PI3-kinase, Akt/protein kinase B (PKB), FoxO1, and other downstream mediators, has been extensively investigated. The important role of plasma insulin concentrations and hepatic insulin sensitivity in the control of glucose metabolism in liver has been studied well, too [15, 53]. However, the role of PLD in insulin regulation of glucose uptake and glycogen synthesis in hepatocytes remains not fully clear. We have shown that insulin stimulates the PLD activity in classical target tissues (muscle, liver cells) as well as in the brain and kidney cortex. Insulin-induced PLD activation in the muscle neocortex and hepatocytes was followed by enhanced glucose uptake and glycogen synthesis [20, 54, 55].

Stimulation of adipocyte surface receptors with insulin promotes the translocation of ADP-ribosylation factor (ARF) proteins to the cell membranes and the subsequent activation of PLD [56, 57]. A model of PLD activation by insulin has been proposed [57]. Using HIRcB cells, a Rat-1 fibroblast cell line that overexpresses human insulin receptor, it has been determined that ARF activation can be regulated by specific guanine nucleotide exchange factors, members of the cytohesin/ARF nucleotide-binding site opener (ARNO) family. Insulin, upon binding to its receptor, not only promotes the phosphorylation of IRS1 and the activation of PI3-kinase, but promotes the recruitment of ARNO or ARF-specific guanine nucleotide exchange factors (GEFs) to the plasma membrane. The recruitment of ARF-GEFs to the plasma membrane in insulin-stimulated cells is stabilized by the interactions of their PH domain with polyphosphoinositides generated by the PI3-kinase and is followed by the PLD activation. Our findings that specific inhibitors of PI3-kinase, wortmannin and LY294002, nullified the stimulatory effect of insulin on PLD activity, glucose uptake, and glycogen synthesis in the liver cells suggest that the PI3-kinase plays an important role in the PLD stimulation by insulin and that PI3-kinase-dependent activation of the PLD can be involved in insulin regulation of glucose metabolism in the primary hepatocytes. To clarify the impact of PLD in glucose metabolism regulation in the insulin-stimulated hepatocytes, the cells were pretreated with different inhibitors of PLD/PA pathway.

To prevent the PLD-dependent PA production and accumulation in the insulin-treated hepatocytes, 1-butanol or 2-butanol as control was used in the present work. Culturing of liver cells in the presence of 1-butanol prior to the insulin addition did not change the hormone stimulatory effect on PLD activity and significantly decreased, but not nullified, the induction of glucose uptake and glycogen synthesis by insulin in the hepatocytes, while 2-butanol practically can not be used in the transphosphatidylation reaction, catalyzed by PLD, and therefor can not eliminate PA accumulation and glucose metabolism stimulation in the insulin-treated cells. Moreover, we observed that inhibition of phosphatidate phosphohydrolase and conversion of PA to other insulin signaling mediators, DAG, by propranolol nullified the stimulatory effect of insulin on the PLD activity and glucose uptake by the hepatocytes. The results obtained suggest that the activation of PA phosphohydrolase and DAG production can play an important role in the glucose metabolism regulation in the hepatocytes. However, propranolol could act not only on PA metabolism, but on other components of insulin signaling pathways which complicates an interpretation of results when using this drug in signal transduction studies. Taking into consideration the fact that propranolol inhibits not only PA phosphohydrolase, but PKC [58] and PKC-dependent PLD activity [59, 60], too, it can not be ruled out that drug, inhibiting PKC, can reduce stimulation of PLD by insulin in rat hepatocytes.

Using mouse hepatocytes overexpressing key enzymes, that catalyze the production of PA (diacylglycerol kinase-*θ*, PLD1/2, etc.), it has been shown that PA accumulation, not related to its source, inhibited insulin signaling [61]. PA is accumulated usually in the cells treated by propranolol due to the PA phosphohydrolase inhibition. Moreover, propranolol, as demonstrated in our work, blocked the glucose metabolism induced by insulin. Taken together, these results suggest that the oversupply of PA is precisely the main reason for glucose metabolism deregulation by propranolol, but not the decreased DAG production.

It is well documented that not only propranolol but ethanol and butanol could act not only on PLD and PA metabolism, but on other components of insulin signaling pathways, such as PKCs and IR [58, 62, 63]. Butanol-1, as well as PI3-kinase inhibitor, LY294002, reduced significantly Akt phosphorylation on its serine 473 and glucose transporter 1 translocation to the membranes, stimulated by PLD, in insulin responsive cells, C2C12 myoblasts [64]. Butanol-1 significantly reduced glucose uptake and aPKC (PKC*ξ*/*λ*) activity during the stimulation of glucose transport and PLD in muscles [65].

It has been found that 1-butanol blocked glucose-stimulated insulin release but did so without inhibiting production of PA, whereas the PLD1/2 inhibitor, FIPI, blocked PA production but had a little effect on insulin release [34]. These data raise the possibility that PBUT functions not as an inert lipid, but rather as a potent inhibitor of the secretory pathway. The link between the PLD and physiological state of the cell can be defined by means of other approaches, such as RNAi-mediated downregulation of individual isoforms of PLD [66]. However, the RNAi-mediated effects took place over hours and days, raising the possibility of secondary effects of the PLD knockdowns, whereas the specific inhibitors permit analysis of acute inhibition of PLD activity.

The present study demonstrated for the first time that specific inhibitor halopemide nullified the insulin stimulatory effect on PLD activity and significantly reduced the induction of the [^3^H]glucose uptake and [^14^C]glycogen synthesis by insulin in the primary hepatocytes. Halopemide and all of the halopemide analogues are described as dual PLD1/2 inhibitors [37]. Halopemide analog, FIPI, widely used for PLD inhibition, was not toxic for cells and did not change the Akt and ERK phosphorylation in both the nonstimulated as well as in the serum-stimulated cells expressing wild-type PLD1 and PLD2 [67]. In addition, the PLD inhibitor FIPI did not alter PLD distribution in the cells and PIP2 availability on the plasma membrane in PLD1- and PLD2-overexpressing cells. Thus, it becomes evident that any mechanisms of halopemide-derived inhibitor other than direct inhibition of the PLD catalytic activity have not been identified. In the HepG2 cells, as well as in the primarily derived human hepatocytes silencing PLD2 by siPLD2 or by halopemide analog, FIPI, was associated with a significant reduction in atypical PKC*ξ* [68]. Moreover, it has been demonstrated that PA can activate PKC*ξ*/*λ* in the skeletal muscles [65]. So it must not be ruled out that insulin activating PLD in the rat hepatocytes can lead to the PA accumulation and to subsequent aPKC activation. However, we should note that excessively activated aPKC can promote insulin resistance. In contrast to the pathophysiological conditions, insulin-induced PLD-dependent production of PA in normal cells is followed by its rapid degradation and conversion to lysoPA, DAG, or phospholipids. This conversion appears to prevent cell from PA oversupply and subsequent overactivation of aPKC.

The PLD1 and PLD2 are both involved in insulin-dependent PLD signaling [3, 13, 31, 32]. The overexpression of a catalytically inactive variant of PLD2 in the HIRcB cells blocks insulin-induced activation of PLD and MAPK phosphorylation, whereas a catalytically inactive PLD1 does not [31]. These results suggest that PLD2 is the main type of phospholipase involved in insulin-dependent PLD signaling. In contrast, in human embryonic kidney-293 cells the insulin-induced PLD1 and PLD2 activity has been found [3]. Moreover, the colocalization of PLD1, but not PLD2, with Glut4 in intracellular membranes of 3T3-L1 adipocytes was observed [32]. Enhancement of Glut4 translocation upon microinjection of purified PLD into cultured adipocytes treated with insulin has been demonstrated. The PLD1 activation plays a rate-limiting step in the process of insulin-stimulated fusion of Glut4-containing storage vesicles into the plasma membrane and increase of [^3^H]glucose uptake by adipocytes [13]. Different types of Gluts [19] and Glut4 among them [69, 70] are expressed in the liver and liver cells. Together these results and findings reported here suggest the involvement of PLD in Glut-dependent glucose metabolism regulation in the insulin-stimulated hepatocytes. Other experiments are needed to clarify this process.

Sphingolipid ceramide is a well known inhibitor of the insulin signaling in insulin-sensitive cells. Because of Akt/PKB inhibition due to induction of dephosphorylation of the protein kinase and prevention of its translocation into the plasma membrane ceramide suppresses the glycogen synthesis. It has been demonstrated that ceramide increased phosphorylation of Thr-563/560-PKC*ξ*/*λ*, thus imitating effects of insulin and product of PI3-kinase, PIP3, on aPKC in mouse liver [71]. Moreover, the C6-ceramide (but not the inactive analog, dihydro-C6-ceramide) induced PKC*ξ* activity and also caused a selective increase in the association between Akt and PKC*ξ* [72]. C6-ceramide, as well as C2-ceramide, did not alter Akt-P in nonstimulated cells and cells expressing dominant negative PKC*ξ* and decreased Akt phosphorylation in the cells, stimulated by platelet derived growth factor. These data suggest that ceramide-mediated overactivation of PKC*ξ* leads to diminishing the Akt phosphorylation.

PLD is an important ceramide target in cells. Ceramide inhibits PLD, competing with phosphoinositide 4,5-bisphosphate for the catalytic site of the enzyme [73] and blocking translocation of ARF protein and PKC to cellular membranes [74]. Moreover, the ceramide can destruct the lipid raft structure and thus inhibit the PLD activity [75] and decrease the PLD expression in the stimulated cells [76]. Recently we have demonstrated that the short-long treatment of hepatocytes, isolated from the livers of adult rats, with D-erythro-N-acetylsphingosine (C2-ceramide) led to the old age-like increase of long-chain ceramides contents and sustained decrease of cells response to insulin action [20]. Similar effects were obtained under treatment of muscle and brain cortex tissues with C2-ceramides [54, 55, 77]. Ceramide accumulation and resistance of C2-ceramide-treated hepatocytes to insulin action were reversible [20, 21]. Addition of inhibitor of sphingolipid synthesis* de novo*, myriocin, nullified the C2-ceramide effect on ceramide accumulation and hepatocytes resistance to the insulin action. It is worth noting that exogenous palmitic acid imitates the C2-ceramide action on primary hepatocytes and under myriocin action palmitic acid-induced insulin resistance of liver cells can be abolished. The results obtained clearly demonstrated that activation of* de novo* synthesis of ceramides contributes to a decrease in the cell sensitivity to insulin. The C6-ceramide, as shown in the present work, reduced the basal PLD activity, glucose uptake, and glycogen synthesis in hepatocytes and abolished the stimulatory effect of insulin on the PLD activity and glucose metabolism in liver cells, while doxorubicin, increasing the ceramide content in the cells, nullified stimulatory effect of insulin on PLD activity and glucose metabolism in the primary hepatocytes. Decrease of ceramide contents in the doxorubicin-treated cells by the mixture of specific inhibitors of ceramide synthesis (myriocin) and degradation (imipramine, GW4869) normalized the hepatocytes response to insulin action. It is also demonstrated that inhibition of PLD activity by specific enzyme inhibitor, halopemide, prior to the pharmacological downregulation of ceramide level in the cells decreased the insulin-stimulated glucose uptake and glycogen synthesis in the hepatocytes. These results confirm and extend data on the significant role of PLD in the regulation of glucose metabolism in insulin-stimulated primary hepatocytes.

Previously, it has been shown that flavones are potent modulators of both SM turnover and insulin signaling in the liver cells. Apigenin, AP7Glu, and other flavonoids of* Chamomilla recutita*, suppressing neutral SMase, prevent ceramide production and accumulation in the CCl4- and ethanol-treated young rat hepatocytes [50]. AP7Glu, as well as LU7Glu, abolishes ceramide level elevation in the hepatocytes of old rats mainly via neutral SMase inhibition [49]. However, both drugs, AP7Glu and LU7Glu, did not alter ceramide content and SM turnover in the hepatocytes isolated from livers of young 3-month-old rats [49, 50]. Using human hepatoma (HepG2), adipose and epithelial cells, it was found that flavones, apigenin and luteolin, are responsible for the inhibition of Akt phosphorylation/activity and Akt signaling pathway [47, 48, 78]. LU7Glu was as potent as luteolin [79]. Apigenin and luteolin also appeared to inhibit insulin-stimulated activation of a downstream effector of PI3K, Akt, and to suppress insulin-stimulated translocation of a Glut4 into the plasma membrane and 2-deoxy-D[1-^3^H]glucose uptake by mouse adipose cells [47]. Apigenin and luteolin, added after insulin within 15 min, reversed the Akt phosphorylation during the following 30 min and reduced the basal phosphorylation of Akt in the human hepatocellular carcinoma HepG2 cells [48]. We have clarified that AP7Glu and LU7Glu are potent suppressors of insulin-stimulated PLD and glucose metabolism in the liver cells. Inhibitory effect of AP7Glu and LU7Glu on PLD and glucose turnover is not dependent on flavones action on insulin receptor. Moreover, flavones action on insulin signaling is not dependent on ceramide accumulation in the hepatocytes [49, 50]. All together these findings do suggest that PLD activation in the insulin-stimulated hepatocytes lie downstream of Akt. Further investigations with use of specific inhibitors of key participants of insulin signaling (Akt, aPKC, etc.) are required to confirm these conclusions.

## 5. Conclusion

The present study indicates that insulin activates the PC-specific PLD activity in the classical insulin responsive tissues (liver, muscles) as well as in the brain and kidney cortex. Activation of PLD in the insulin-stimulated primary hepatocytes is associated with increase of glucose uptake and glycogen synthesis. Blocking the PLD-dependent PA accumulation by 1-butanol prevents stimulation of glucose metabolism by insulin in the liver cells, while inhibition of the PA conversion to other insulin signaling mediators, DAG, by propranolol nullified stimulatory insulin effect on the PLD activity and glucose uptake in the hepatocytes. Moreover, the fact that insulin-induced activation of PLD/PA pathway can be blocked by specific inhibitors of PI3-kinase demonstrates that PLD is activated by insulin downstream of PI3-kinase. AP7Glu/LU7Glu-induced alterations in insulin-stimulated PLD activity suggest that PLD activation lies downstream of Akt, too. The regulation of PLD and glucose metabolism by insulin in the primary hepatocytes is sensitive to a well known antagonist of PI3-kinase/Akt-dependent signaling pathways, ceramide. Activation of PLD as well as glucose metabolism in the insulin-stimulated cells can be significantly reduced by using the exogenous C6-ceramide and doxorubicin-induced endogenous long-chain ceramides accumulation. Normalization of the ceramide content in the doxorubicin-treated hepatocytes by specific inhibitors of sphingolipid turnover improves the cells response to insulin action. Taking into account the fact that ceramide, as well as 1-butanol and propranolol, can act not only on PLD/PA pathway but on other participants of insulin signaling, too, the specific inhibitor of PLD, halopemide, was used to clarify the role of PLD in the regulation of glucose metabolism by insulin. Halopemide suppresses the PLD activity and significantly reduces stimulation of glucose uptake and glycogen synthesis by insulin in hepatocytes. Besides this, halopemide nullified the stimulatory effect of insulin in the liver cells treated with doxorubicin + total sphingolipid turnover inhibitors. Thus, it is evident that PLD plays an important role in the insulin signaling. The PLD activated downstream of PI3-kinase/Akt is highly sensitive to ceramide content in the primary hepatocytes. The key role of PLD in the development of drug-induced insulin resistance has been demonstrated in the liver cells. Pharmacological modulation of PLD activity, as well as ceramides content in the cells, can be a useful tool for manipulating the hepatocytes sensitivity to insulin action.
